# A new model of endotracheal tube biofilm identifies combinations of matrix-degrading enzymes and antimicrobials able to eradicate biofilms of pathogens that cause ventilator-associated pneumonia

**DOI:** 10.1099/mic.0.001480

**Published:** 2024-08-01

**Authors:** Dean Walsh, Chris Parmenter, Saskia E. Bakker, Trevor Lithgow, Ana Traven, Freya Harrison

**Affiliations:** 1 School of Life Sciences, University of Warwick, Coventry, UK; 2 Nanoscale and Microscale Research Centre, University of Nottingham, Nottingham, UK; 3 Department of Biochemistry and Molecular Biology, Infection Program, Biomedicine Discovery Institute, Monash University, Clayton 3800, Victoria, Australia; 4 Center To Impact AMR, Monash University, Clayton 3800, Victoria, Australia

**Keywords:** antimicrobial resistance, antimicrobial tolerance, biofilm, endotracheal tube, hospital-acquired infection, infection

## Abstract

Ventilator-associated pneumonia is defined as pneumonia that develops in a patient who has been on mechanical ventilation for more than 48 hours through an endotracheal tube. It is caused by biofilm formation on the indwelling tube, which introduces pathogenic microbes such as *Pseudomonas aeruginosa*, *Klebsiella pneumoniae* and *Candida albicans* into the patient’s lower airways. Currently, there is a lack of accurate *in vitro* models of ventilator-associated pneumonia development. This greatly limits our understanding of how the in-host environment alters pathogen physiology and the efficacy of ventilator-associated pneumonia prevention or treatment strategies. Here, we showcase a reproducible model that simulates the biofilm formation of these pathogens in a host-mimicking environment and demonstrate that the biofilm matrix produced differs from that observed in standard laboratory growth medium. In our model, pathogens are grown on endotracheal tube segments in the presence of a novel synthetic ventilated airway mucus medium that simulates the in-host environment. Matrix-degrading enzymes and cryo-scanning electron microscopy were employed to characterize the system in terms of biofilm matrix composition and structure, as compared to standard laboratory growth medium. As seen in patients, the biofilms of ventilator-associated pneumonia pathogens in our model either required very high concentrations of antimicrobials for eradication or could not be eradicated. However, combining matrix-degrading enzymes with antimicrobials greatly improved the biofilm eradication of all pathogens. Our *in vitro* endotracheal tube model informs on fundamental microbiology in the ventilator-associated pneumonia context and has broad applicability as a screening platform for antibiofilm measures including the use of matrix-degrading enzymes as antimicrobial adjuvants.

## Data Summary

Four supplementary figures and four supplementary tables are available at 10.6084/m9.figshare.26305429 [1].

## Importance

The incidence of ventilator-associated pneumonia in mechanically ventilated patients is between 5 and 40 %, increasing to 50–80 % in patients suffering from coronavirus disease 2019 (COVID-19). The mortality rate of ventilator-associated pneumonia patients can reach 45%. Treatment of the endotracheal tube biofilms that cause ventilator-associated pneumonia is extremely challenging, with causative organisms able to persist in the endotracheal tube biofilm despite appropriate antimicrobial treatment in 56% of ventilator-associated pneumonia patients. Flawed antimicrobial susceptibility testing often means that ventilator-associated pneumonia pathogens are insufficiently treated, resulting in patients experiencing ventilator-associated pneumonia recurrence. Here we present an *in vitro* endotracheal tube biofilm model that recapitulates key aspects of endotracheal tube biofilms, including dense biofilm growth and elevated antimicrobial tolerance. Thus, our biofilm model can be used as a ventilated airway simulating environment, aiding in the development of anti-ventilator-associated pneumonia therapies and antimicrobial endotracheal tubes that can 1 day improve the clinical outcomes of mechanically ventilated patients.

## Introduction

Biofilms are congregations of microbial cells coated in a self-produced exopolymeric matrix consisting of extracellular DNA, exopolysaccharides, matrix proteins and lipids; the biofilm aggregate is often surface-attached but can be free-floating [2]. The microbes ensconced in a biofilm are extremely difficult to treat with antimicrobial therapy due to: (i) antimicrobial diffusion being impeded by the biofilm matrix [3,7], (ii) nutritional starvation reduces the metabolic activity of cells within biofilms, protecting against antimicrobials that target active cellular processes [8,9], (iii) an increased population of persister cells within biofilms [10,11] and (iv) hypermutability and horizontal gene transfer allow for the acquisition of genetic mechanisms of antimicrobial resistance [11,13].

Medical device-related infections account for a quarter of all nosocomial infections [14]. In the case of critically ill patients intubated with endotracheal tubing to facilitate mechanical ventilation of the airways, intubation increases the risk of pulmonary infection up to 20-fold [15,16]. Intubation compromises the local immune response, prevents the cough reflex, impedes mucociliary clearance of entrapped microorganisms and damages the tracheal epithelium. Following intubation, oropharyngeal flora and nosocomial pathogens colonize the trachea and form biofilms on the endotracheal tubing. Furthermore, the accumulation of contaminated secretions above the endotracheal tube cuff leads to microaspiration of these secretions, seeding biofilm in other regions of the endotracheal tube and airways, potentially resulting in ventilator-associated pneumonia (VAP) [17,19].

Many oral commensal and hospital-acquired pathogen species can form monospecies or polymicrobial biofilms on endotracheal tubes *in vivo* [20,21]. A previous study found that the pathogens found in the lung were identical to those constituting endotracheal tube biofilms in 70% of VAP patients [22]. *Staphylococcus aureus*, *Klebsiella pneumoniae*, *Acinetobacter baumannii* and *Pseudomonas aeruginosa* are among the bacterial pathogens most commonly associated with VAP, particularly with late-onset disease in which multidrug-resistant strains become prevalent [23,25]. The fungi *Candida albicans*, an oral commensal and opportunistic pathogen, rarely causes VAP but frequently colonizes endotracheal tubes [20,23, 26, 27].

Eradication of endotracheal tube biofilms is extremely challenging, with one study finding causative organisms persisting on endotracheal tubes despite appropriate antimicrobial treatment in 56% of VAP patients [26]. Furthermore, VAP recurrence occurs in 26% of patients, with an average 2–5 recurrences per patient; this is often due to failed treatment of the initial pathogen [28]. Endotracheal tubes coated with silver or a combination of silver and other metals have been shown to reduce microbial colonization in lab studies and have been approved for clinical use; other innovative antimicrobial tube coatings have not progressed beyond pre-clinical testing [29]. Silver-coated endotracheal tubes have been shown to delay the onset of VAP or reduce its incidence as defined by specific diagnostic criteria; however, there is little evidence to suggest they reduce crucial patient outcomes including ventilator-associated events that fall short of a VAP diagnosis, hospital stay, mechanical ventilation duration or patient mortality. This suggests that they have limited ability to prevent microbial colonization *in vivo* and makes it difficult for clinicians to justify the higher costs of these tubes [30,31], demonstrating the need for further innovation in endotracheal tube development.

Relapses in infection can often be attributed to flawed antimicrobial susceptibility testing [32,35]. Conventional antimicrobial susceptibility testing methods cannot always predict the efficacy of antibiotic treatment *in vivo* [32] and do not factor in biofilm or polymicrobial communities [33,35]. To bridge this disconnect between conventional antimicrobial susceptibility testing and the *in vivo* environment, researchers have developed biofilm models that attempt to capture the infection environment and increase diagnostic accuracy [36]. These include Calgary devices in which bacteria can form biofilms on plastic pegs [37,38], however, these methods do not provide much improvement on standard antimicrobial susceptibility testing [39]. More recent biofilm models, such as our *ex vivo* model of the cystic fibrosis airways, which combines pig lung tissue with synthetic cystic fibrosis mucus, produce biofilms with gene expression profiles that resemble those observed in patient sputum [3,40,50]. The *in vitro* and *ex vivo* models developed to better mimic the airways of individuals with cystic fibrosis [51] stand in stark contrast to VAP, where animal models are relied on for the study of the disease [52,53].

**Fig. 1.**
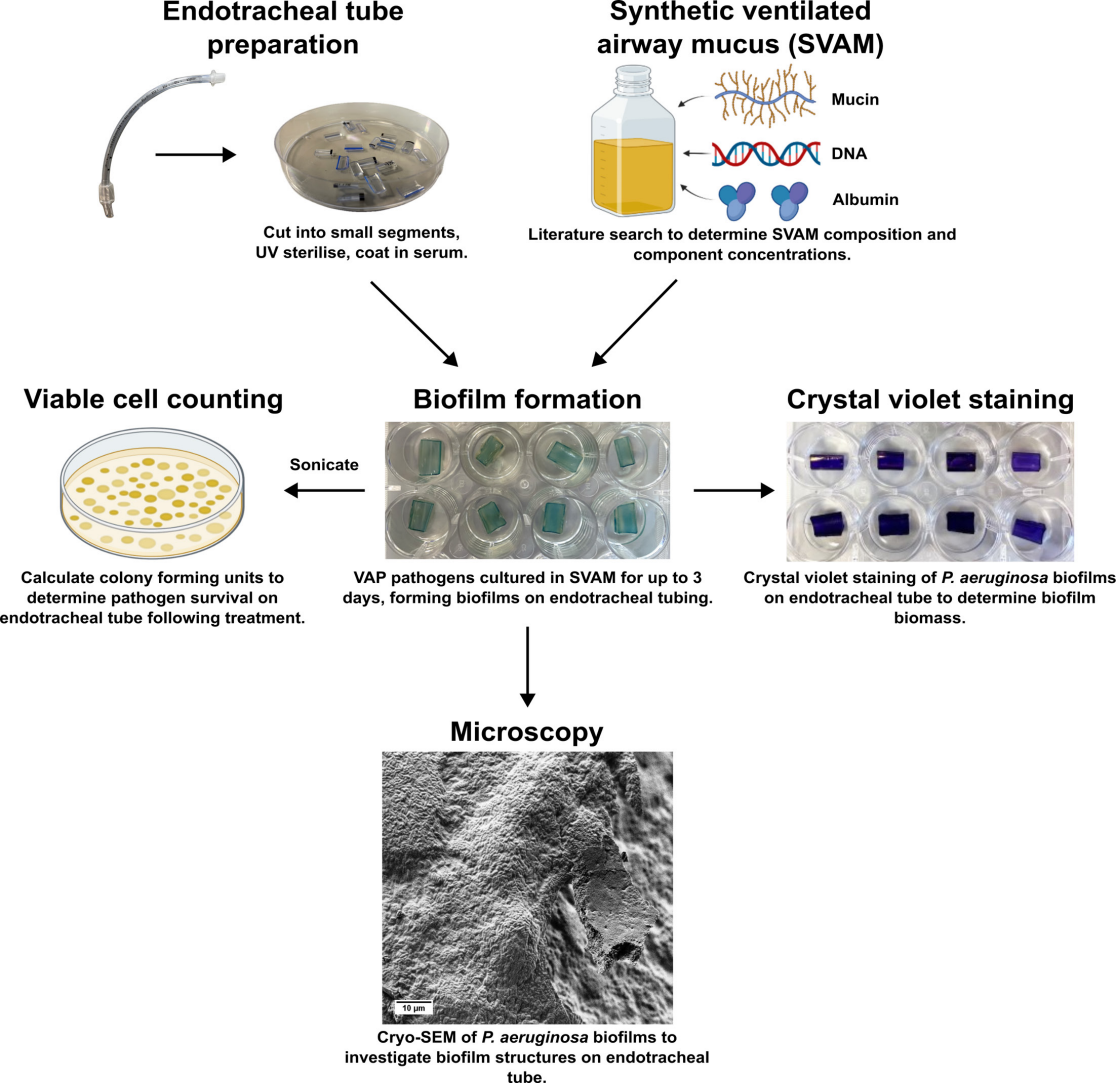
Schematic and applications of the *in vitro* endotracheal tube (IVETT) biofilm model. *P. aeruginosa* biofilms are shown as an example to illustrate the model. Figure created with BioRender.com.

Here, we showcase a novel *in vitro* biofilm model featuring a newly developed synthetic ventilated airway mucus (SVAM) growth medium and serum-coated endotracheal tubes (Fig. 1). This model simulates the nutritional and material environment that pathogens are exposed to as they colonize patient airways. We found that biofilms of *P. aeruginosa*, *K. pneumoniae* and * C. albicans* cultured in this model were much harder to eradicate with antimicrobial treatment compared to cultures in standard antimicrobial susceptibility testing conditions. Furthermore, cryo-scanning electron microscopy (cryo-SEM) and enzymatic degradation assays revealed that biofilm structure and matrix composition differed depending on the growth environment. We then used our model to screen combinations of antibiotics and matrix-degrading enzymes against biofilms of VAP pathogens. This combination treatment resulted in improved biofilm eradication. Collectively, this study illustrates how a tailored model of endotracheal tube biofilm may be used to test emerging therapies designed to prevent biofilm formation and the onset of VAP in mechanically ventilated patients.

## Methods

### Strains and culture conditions

Cultures of *P. aeruginosa* PA14 and *K. pneumoniae* B5055 were grown aerobically at 37 °C overnight on lysogeny broth (LB) agar plates for solid culture, and overnight in LB broth at 37 °C with shaking at 150 r.p.m. for liquid culture. *C. albicans* SC5314 solid cultures were grown aerobically overnight on yeast extract peptone dextrose (YPD) agar plates at 37 °C, while liquid cultures were grown overnight in YPD broth at 37 °C with shaking at 150 r.p.m.

### SVAM medium

Individual stocks of 9.6 mg ml^−1^ salmon sperm DNA, 17 mM Fe(II)SO_4_·7H_2_O, 95 mM ZnSO_4_·7H_2_O, 9.5 mM CuSO_4_·5H_2_O, 4 mg ml^−1^ sialic acid, and 100 mg ml^−1^ human lysozyme were filter sterilized (0.22 µm, PES filter). A 20 mg ml^−1^ stock of type III porcine gastric mucin was prepared and sterilized by autoclaving. Human lysozyme stock was stored at −20 °C, and all other stocks were stored at 4 °C. The preparation of the SVAM base stock is detailed in Table 1. The preparation of the completed SVAM medium, which is prepared fresh in aseptic conditions immediately prior to each experiment, is detailed in Table 2. The final concentrations of components in stocks and in the final SVAM medium are shown in Table S1 and further details and suppliers of reagents are shown in Table S2, both available in the Supplementary Material.

**Table 1.** Components and method for preparing the SVAM base stock

Component	Amount required 500 ml SVAM base stock	Protocol
**CaCl_2_**	0.236 g	Add each component individually in the order listed to 480 ml of dH_2_O, ensuring each component is fully dissolved before adding the next component. Add NaOH to pH 6.7–6.8. Add dH_2_O to give a final volume of 500 ml. Filter, sterilize (0.22 µm, PES filter) and store at 4 °C.
**Bovine serum albumin**	10.5 g	
**NaCl**	3.33 g	
**Na_2_HPO_4_**	0.86 g	
**NaH_2_PO_4_**	0.206 g	
**K_2_SO_4_**	1.087 g	
**KCl**	0.131 g	
**Casamino acids**	7.5 g	
**MOPS**	3.139 g	
**MgCl_2_·6H_2_O**	0.725 g	
**Glucose**	1.08 g	
**DPTA**	0.009 g	
**GlcNAc**	5.25 g	
**Fe(II)SO_4_·7H_2_O**	1.5 ml of 17 mM stock	
**ZnSO_4_·7H_2_O**	1.5 ml of 95 mM stock	
**CuSO_4_·5H_2_O**	1.5 ml of 9.5 mM stock	
**Sialic acid**	1.5 ml of 4 mg ml^−1^ stock	

**Table 2.** Preparation of the final SVAM medium, prepared fresh for each experiment

Component	Amount required for 30 ml of completed SVAM medium	Comments
**SVAM base stock (filter–sterile**)	10 ml	SVAM base stock always makes up 33.3% of the final volume.
**9.6 mg ml^−1^ salmon sperm DNA stock (filter–sterile**)	3 ml	DNA stock always makes up 10% of the final volume
**20 mg ml^−1^ porcine stomach mucin (type III) stock (autoclaved**)	15 ml	Mucin stock always makes up 50% of the final volume.
**100 mg ml^−1^ lysozyme stock (filter sterile**)	15 µl	Add lysozyme to a final concentration of 48 µg ml^−1^.
**NaOH**	Add until a final pH of 6.7–6.8 is reached.	Aseptically check pH.
**dH_2_O**	Add until a 30 ml final volume is achieved.	

### Antimicrobial susceptibility testing using standard platforms

For antimicrobial susceptibility experiments, *P. aeruginosa* and *K. pneumoniae* were grown in cation-adjusted Mueller Hinton broth (caMHB) (Sigma-Aldrich), while *C. albicans* was grown in RPMI 1640 (Gibco) supplemented with 2% (w/v) glucose. All species were also grown in SVAM medium. Relevant media were inoculated with *P. aeruginosa*, *K. pneumoniae* or *C. albicans* to a final OD_600nm_ of 0.05 into 96-well plates (Corning, 3596). *P. aeruginosa* was tested against gentamicin, *K. pneumoniae* against colistin and *C. albicans* against amphotericin B. All organisms were grown aerobically throughout standard antimicrobial susceptibility testing. All antimicrobials were tested in the range of 0.0625–256 µg ml^−1^. For planktonic cultures, MICs were determined by broth microdilution in accordance with European Committee on Antimicrobial Susceptibility Testing guidelines (EUCAST: www.eucast.org). For biofilms, following the same inoculation procedure described above, minimum biofilm eradication concentrations (MBEC) were determined using a peg lid Calgary biofilm device [peg lid: Nunc (445497), plate: Nunc (269787)] as previously described [37].

### Antimicrobial susceptibility testing in the *in vitro* endotracheal tube (IVETT) biofilm model

Under aseptic conditions, endotracheal tubes (siliconized PVC, cuffed, 8 mm, IMS Euro) were cut into 1 cm rings. Each ring was then cut into six equal segments. Segments were sterilized under short-wave UV light (Carlton Germicidal Cabinet) for 10 min. FBS was aseptically poured over the endotracheal tube segments until immersed, and the segments were sealed in a petri dish and left overnight at 4 °C to become coated in serum proteins.

Serum-coated endotracheal tube segments were added to the wells of 24 well plates (Corning Costar, CLS3527). Endotracheal tube segments were immersed in 0.5 ml of SVAM medium and inoculated with 0.05 OD_600_ of relevant microorganisms. The plates were then incubated at 37 °C, 5 % CO_2_ for 48 h to allow biofilm formation. Following incubation, biofilm-coated endotracheal tube segments were transferred to a fresh SVAM medium containing relevant antimicrobials. This transfer simulates the periodic removal of secretions from the airways of ventilated patients and the build-up of fresh secretions. Antimicrobial-exposed biofilms were incubated for a further 24 h at 37 °C and 5 % CO_2_. Biofilms were transferred to fresh plates, washed with 1 ml of PBS, sonicated at 50 Hz (Grant XUBA1 sonicating water bath) for 15 min and then scraped with sterile pipette tips to remove biofilm. Suspensions were plated onto relevant growth medium, and c.f.u./ml was calculated to determine the viability of endotracheal tube biofilm.

### Cryo-SEM of endotracheal tube biofilms

*P. aeruginosa*, *K. pneumoniae* and *C. albicans* were grown on serum-coated endotracheal tubes in either SVAM and LB (*P. aeruginosa* and *K. pneumoniae*) or SVAM and YPD (*C. albicans*), for 72 h at 37 °C with 5 % CO_2_. Biofilms were fixed for 2 h in 2 % paraformaldehyde in PBS. Imaging was carried out using a Zeiss Crossbeam 550 focused ion beam-SEM equipped with a Quorum 3010 cryo sample preparation system. Following cryopreparation in slushy nitrogen, biofilm samples were sublimated for 5 min at −90 °C and coated with platinum for 1 min at a current of 10 mA. Endotracheal tube biofilm samples were tilted at 30°, the accelerating voltage of the SEM was maintained at 2 kV and the working distance was maintained at approximately 4–5 mm for each image.

### Susceptibility of endotracheal tube biofilms to matrix-degrading enzymes

Serum-coated endotracheal tube segments were added to the wells of 24 well plates and immersed in 0.5 ml of LB (*P. aeruginosa and K. pneumoniae*), YPD (*C. albicans*) or SVAM, inoculated with 0.05 OD_600_ of relevant micro-organisms. Plates were incubated at 37 °C with 5% CO_2_ for 48 h to allow biofilm formation. Following incubation, biofilm-coated endotracheal tubes were transferred to fresh growth medium containing either 100 µg ml^−1^ DNase I, 1 mg ml^−1^ proteinase K or 10% (w/v) glycoside hydrolases (GH) [5% (w/v) cellulase and 5% (w/v) α-amylase]. Further details on enzyme activity and suppliers are found in Table S2. Biofilms were then incubated for a further 24 h at 37 °C and 5% CO_2_.

Endotracheal tube segments were removed from wells and immersed in a 0.05% (v/v) crystal violet solution for 15 min, washed in PBS and left to dry for 30 min in a laminar flow cabinet. Endotracheal tubes were then immersed in 30% acetic acid for 15 min to solubilize crystal violet. Biomass was determined by reading absorbance at 550 nm.

### Testing antimicrobial and matrix-degrading enzyme combinations in the IVETT biofilm model

Serum-coated endotracheal tube segments were added to the wells of 24 well plates and immersed in 0.5 ml of SVAM medium, inoculated with 0.05 OD_600_ of relevant microorganism. Plates were incubated at 37 °C with 5 % CO_2_ for 48 h to allow biofilm formation. Following incubation, biofilm-coated endotracheal tubes were transferred to fresh SVAM medium containing relevant antimicrobials. Relevant wells were also supplemented with enzymes, as described above. Biofilms were incubated for a further 24 h at 37 °C and 5 % CO_2_. Endotracheal tubes were transferred to fresh plates, washed with 1 ml of PBS, sonicated for 15 min and scraped with sterile pipette tips to remove biofilm. Following the removal of endotracheal tubes from treatment wells and into wells of PBS, c.f.u./ml was determined. c.f.u./ml from the endotracheal tubes determines the viability of biofilm still attached to the endotracheal tube. Total c.f.u./ml was calculated from the sum of the dispersed and biofilm c.f.u.s.

### Data analysis

Raw data are provided in the Data Supplement. Data presentation and statistical analyses were conducted in GraphPad Prism 10. Two-way ANOVAs with Dunnett’s multiple comparisons tests were carried out for biofilm dispersal experiments, with *P* < 0.05 considered significant.

## Results

### SVAM medium increases the MIC and MBEC of clinically relevant antibiotics against VAP pathogens

Gentamicin and colistin were chosen to treat *P. aeruginosa* and *K. pneumoniae*, respectively, as both antibiotics are frequently used to treat multidrug-resistant Gram-negative bacteria [54,55] and carbapenem-resistant VAP infections [54,56]. Amphotericin B was used for *C. albicans* treatment due to its use in airway decolonization of *C. albicans* in mechanically ventilated patients, which is known to reduce VAP incidence and improve prognosis [57].

Although not as well characterized as cystic fibrosis sputum, existing literature does suggest that mucus in the ventilated airways is a distinct growth environment that necessitates a bespoke growth medium. These distinctions include much higher mucin and glucose concentrations compared to those in cystic fibrosis sputum [58,61]. The SVAM medium recipe was developed following an extensive review of existing literature [62]. The effect of each SVAM component on the growth and biofilm formation of * P. aeruginosa*, *K. pneumoniae* and *C. albicans* is shown in Figs S1–S3 in the Supplementary Material, along with accompanying Supplementary Methods.

In a standard broth microdilution assay, the culture medium used affected the concentration of antimicrobials required to inhibit the growth of all species investigated. In the medium used in accordance with EUCAST standards (caMHB for *P. aeruginosa* and *C. albicans* and RPMI glucose for *C. albicans*), all species were more susceptible to their respective antimicrobial treatments than when grown in SVAM (Table 3). When grown in SVAM medium, the gentamicin MIC of *P. aeruginosa* was fourfold higher than in caMHB (caMHB: 4 µg ml^−1^ and SVAM: 16 µg ml^−1^). Both *K. pneumoniae* displayed an eightfold increase in MICs to colistin (caMHB: 1 µg ml^−1^ and SVAM: 8 µg ml^−1^) while *C. albicans* demonstrated a 16-fold MIC increase for amphotericin B (RPMI glucose: 0.125 µg ml^−1^ and SVAM: 2 µg ml^−1^), respectively.

**Table 3.** MICs of clinically relevant antimicrobials against VAP pathogens

Organism	Antimicrobial	Broth microdilution (standard laboratory medium) MIC (µg ml^−1^)	Broth microdilution (SVAM) MIC (µg ml^−1^)
*P. aeruginosa* PA14	Gentamicin	4	16
*K. pneumoniae* B5055	Colistin	1	8
*C. albicans* SC5314	Amphotericin B	0.125	2

In accordance with EUCAST guidelines, the standard laboratory growth medium for *P. aeruginosa* and *K. pneumoniae* is caMHB, while the standard growth medium for *C. albicans* is RPMI 1640 supplemented with 2 % glucose.

**Fig. 2.**
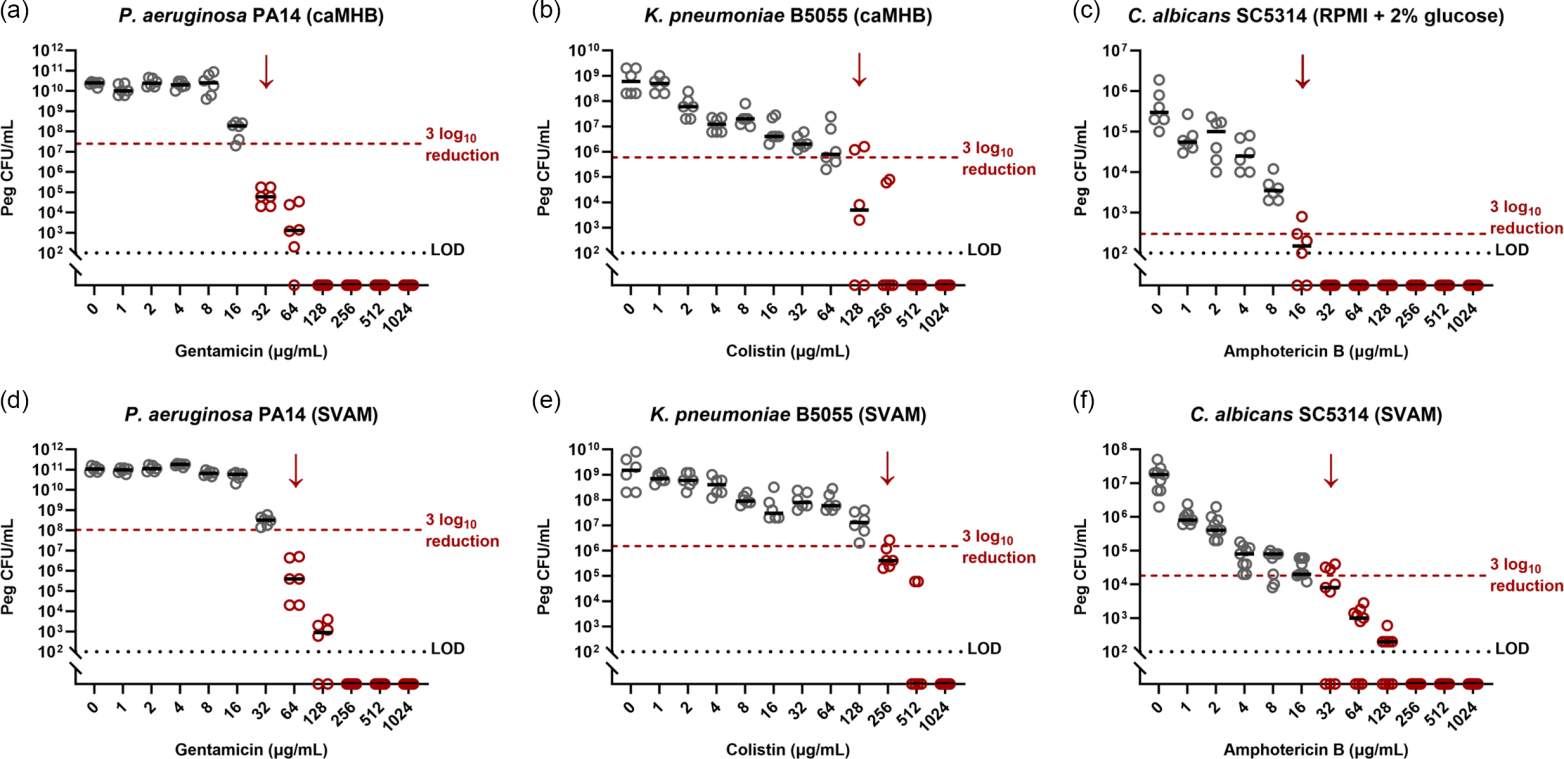
The effect of growth medium on the antimicrobial susceptibility of biofilms grown in a Calgary biofilm device. Biofilms of VAP pathogens were established in peg lid Calgary biofilm devices, in either caMHB or SVAM, for 48 h. Peg lids were then transferred to fresh medium with antimicrobials for a further 24 h. Biofilms were removed from pegs, and c.f.u.s were counted to determine endpoint viability. The MBEC is defined as the antimicrobial concentration needed to achieve an average 3 log_10_ reduction in c.f.u.s relative to untreated controls. (**a**) Endpoint c.f.u.s of *P. aeruginosa* biofilms grown in caMHB. (**b**) Endpoint c.f.u.s of *K. pneumoniae* biofilms grown in caMHB. (**c**) Endpoint c.f.u.s of *C. albicans* biofilms grown in caMHB. (**d**) Endpoint c.f.u.s of *P. aeruginosa* biofilms grown in SVAM. (**e**) Endpoint c.f.u.s of *K. pneumoniae* biofilms grown in SVAM. (**f**) Endpoint c.f.u.s of *C. albicans* biofilms grown in SVAM. The dotted line denotes the 3 log_10_ reduction threshold. A black dotted line denotes the limit of detection (LOD). The red arrow highlights the MBEC, *n* = 3 biological repeats (experiment repeated on different days with fresh starting cultures), with two technical repeats (*P. aeruginosa* and *K. pneumoniae*) or three technical repeats (*C. albicans*) for each biological replicate.

As expected, the antimicrobial concentrations required to eradicate biofilms grown in a peg lid biofilm Calgary device exceeded the MIC required in planktonic culture. MBECs for all pathogens were twofold higher in SVAM than in the EUCAST-dictated medium. *P. aeruginosa* gentamicin MBECs increased from 32 µg ml^−1^ in caMHB (Fig. 2a) to 64 µg ml^−1^ in SVAM (Fig. 2d), * K. pneumoniae* colistin MBECs increased from 128 µg ml^−1^ in caMHB (Fig. 2b) to 256 µg ml^−1^ in SVAM (Fig. 2e) and *C. albicans* amphotericin B MBECs increased from 16 µg ml^−1^ in RPMI glucose (Fig. 2c) to 32 µg ml^−1^ in SVAM (Fig. 2f). Changes in antimicrobial tolerance between either caMHB or RPMI glucose and SVAM do not appear to be linked to growth rate across all species. Growth curves show that both *P. aeruginosa* (Fig. S4A) and *K. pneumoniae* (Fig. S4B) have comparable planktonic growth profiles when grown in either caMHB or SVAM. Alternatively, *C. albicans* displays reduced planktonic growth in SVAM compared to RPMI glucose (Fig. S4C). However, this does not appear to impact the viable cell population of *C. albicans* biofilms, with untreated SVAM-grown *C. albicans* peg lid biofilms actually yielding higher average population sizes (Fig. 2c) compared to untreated RPMI glucose-grown peg lid biofilms (Fig. 2f).

**Fig. 3.**
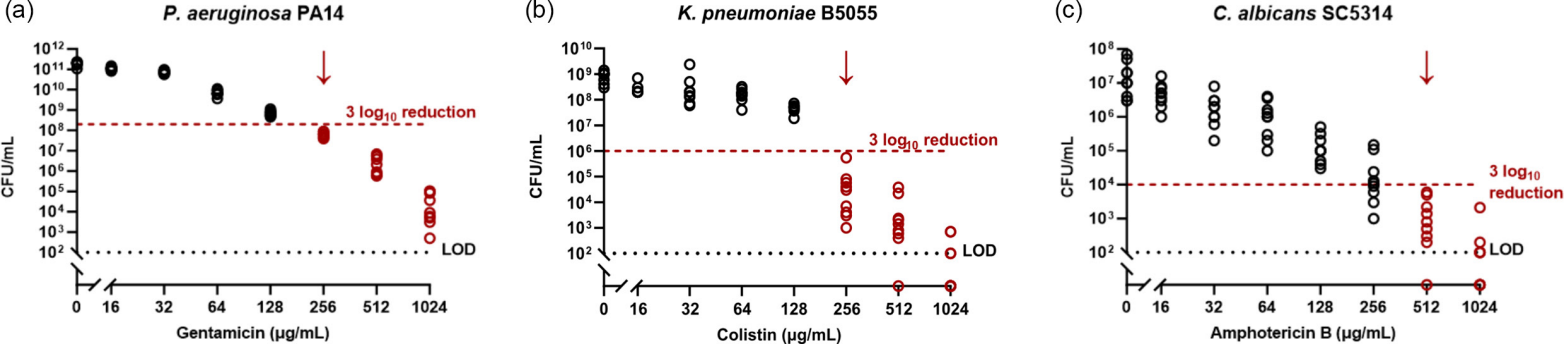
Susceptibility of biofilms to clinically relevant antimicrobials in the IVETT model. VAP pathogens were grown on segments of serum-coated endotracheal tube in SVAM for 48 h to establish biofilms. Endotracheal tubes were then transferred to fresh SVAM with antimicrobials for a further 24 h. Biofilms were removed from endotracheal tube segments, and c.f.u.s were counted to determine endpoint viability. MBECs were defined as the first antimicrobial concentration to achieve an average 3 log_10_ reduction in c.f.u.s relative to untreated controls. (**a**) Endpoint c.f.u.s of *P. aeruginosa* biofilms. (**b**) Endpoint c.f.u.s of *K. pneumoniae* biofilms. (**c**) Endpoint c.f.u.s of *C. albicans* biofilms. The red dotted line denotes the 3 log_10_ reduction threshold. The black dotted line denotes the LOD. The red arrow highlights the MBEC, *n* = 3 biological repeats, with three technical repeats for each biological replicate.

Further increases in antimicrobial tolerance were observed when pathogens formed biofilms on serum-coated endotracheal tubes in SVAM. Relative to the peg lid biofilms, eradication of *P. aeruginosa* (Fig. 3a) and *C. albicans* (Fig. 3c) required fourfold higher concentrations of gentamicin (4× higher; 256 µg ml^−1^) and amphotericin B (16× higher; 512 µg ml^−1^), respectively. However, while the presence of serum-coated endotracheal tubes increased the antimicrobial tolerance of some VAP pathogens, this was not universal, with *K. pneumoniae* displaying no change in colistin susceptibility (256 µg ml^−1^) between the peg lid Calgary device (Fig. 2e) and the endotracheal tube model (Fig. 3b).

### SVAM impacts the structure, matrix composition and susceptibility to matrix-degrading enzymes of biofilms grown on endotracheal tubes

Since biofilms grown in the IVETT model were more tolerant to antimicrobial therapy, we sought to discern whether changes in biofilm structure and matrix composition might explain this. Biofilms of VAP pathogens were grown on endotracheal tubes either in SVAM to mimic the VAP environment or in standard laboratory growth media (LB for *P. aeruginosa* and *K. pneumoniae*; YPD for *C. albicans*) and cryo-SEM was employed to visualize the biofilm ultrastructure.

**Fig. 4.**
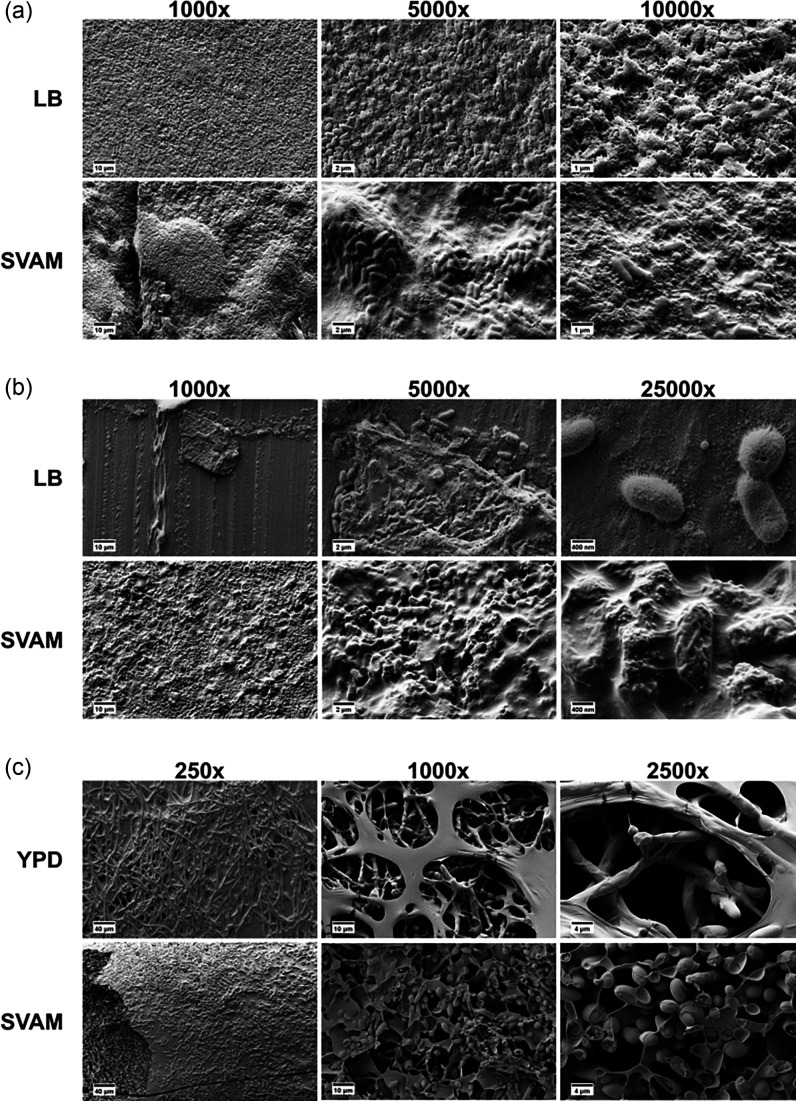
Cryo-SEM shows that the endotracheal tube environment alters the biofilm structure of *P. aeruginosa*, *K. pneumoniae* and *C. albicans*. (**a**) Representative images of *P. aeruginosa* biofilms grown on a serum-coated endotracheal tube, either in LB or SVAM. (**b**) Representative images of *K. pneumoniae* biofilms grown on a serum-coated endotracheal tube, either in LB or SVAM. (**c**) Representative images of *C. albicans* biofilms grown on a serum-coated endotracheal tube, either in YPD or SVAM. Relevant magnifications are shown above each set of images.

Cryo-SEM of *P. aeruginosa* biofilms showed that dense biofilm coats the endotracheal tube in either growth medium. However, * P. aeruginosa* endotracheal tube biofilms grown in LB appeared flat and homogenous, whereas those grown in SVAM had a varied topography, with microcolony structures covering the tube (Fig. 4a). When *K. pneumoniae* biofilms were grown in LB, only small aggregates of biofilm and single cells could be found on the endotracheal tube (Fig. 4b). Conversely, SVAM resulted in * K. pneumoniae* biofilms covered in a thick matrix. Furthermore, *K. pneumoniae* cells in LB-grown biofilms had a smooth surface, whereas those grown in SVAM had a rough cell surface, which is likely due to a thicker coating of capsular polysaccharide. Lastly, YPD-grown endotracheal tube biofilms of *C. albicans* were largely composed of layers of hyphae (Fig. 4c), whereas those grown in SVAM instead favoured yeast cells and pseudohyphae. This was particularly apparent in the images of freeze-fractured *C. albicans* biofilms, showing a biofilm interior composed of many layers of yeast cells and pseudohyphae. *C. albicans* biofilms grown in SVAM were also much thicker than those grown in YPD. All imaged biofilms were grown on serum-coated endotracheal tube segments, indicating that structural differences were solely due to differences in growth medium rather than the presence of serum.

**Fig. 5.**
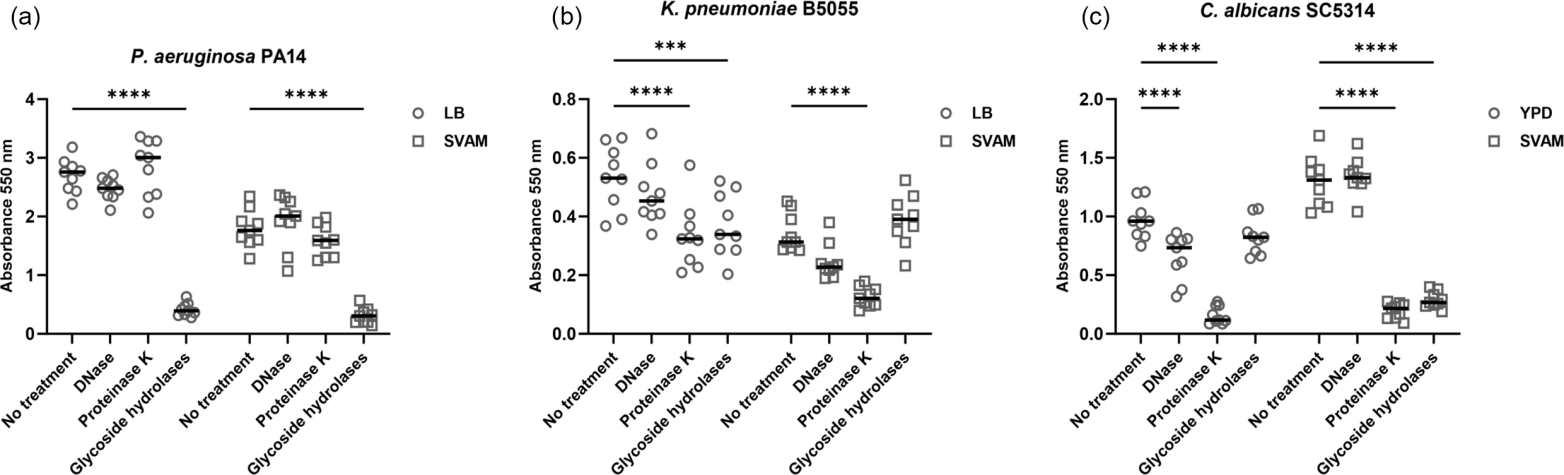
Susceptibility of endotracheal tube biofilms to matrix-degrading enzymes when grown in standard laboratory medium compared to SVAM medium. VAP pathogens were grown on segments of serum-coated endotracheal tube in either LB (*P. aeruginosa* and *K. pneumoniae*), YPD (*C. albicans*) or SVAM for 48 h to establish biofilms. Endotracheal tubes were then transferred to fresh medium and treated with either 100 µg ml^−1^ DNase, 1 mg ml^−1^ proteinase K or 10% GH for a further 24 h. Endotracheal tube biofilms were then stained with crystal violet and absorbance was measured at 550 nm to determine changes in biofilm biomass. (**a**) Biomass of *P. aeruginosa* endotracheal tube biofilms (enzyme: F_3,64_ = 164.6, *P* < 0.0001; medium: F_1_,_64_ = 94.85, *P*<0.0001; interaction: F_3,64_ = 11.27, *P* < 0.0001). (**b**) Biomass of *K. pneumoniae* endotracheal tube biofilms (enzyme: F_3,64_ = 17.46, *P* < 0.0001; medium: F_1_,_64_ = 52.29, *P* < 0.0001; interaction: F_3,64_ = 7.24, *P* = 0.0003). (**c**) Biomass of *C. albicans* endotracheal tube biofilms (enzyme: F_3,64_ = 159.9, *P* < 0.0001; medium: F_1_,_64_ = 13.98, *P* = 0.0004; interaction: F_3,64_ = 56.58, *P* < 0.0001). Statistical significance was determined by a two-way ANOVA with Dunnett’s multiple comparisons test. **** denotes *P* ≤ 0.0001, *** denotes *P* ≤ 0.001 and *n* = 3 biological repeats, with three technical repeats for each biological replicate.

Matrix composition was determined by treating established endotracheal tube biofilms with hydrolytic enzymes. DNase I, proteinase K and GH degrade extracellular DNA, matrix proteins and exopolysaccharides, respectively. Following enzymatic treatment, crystal violet staining was used to detect residual biofilm. Treatment with GH was able to significantly disperse * P. aeruginosa* biofilms grown in either LB or SVAM from the endotracheal tube segment (Fig. 5a). Conversely, *K. pneumoniae* (Fig. 5b) and *C. albicans* (Fig. 5c) biofilms demonstrated differences in matrix-degrading enzyme susceptibilities when grown in different media. Biofilms of *K. pneumoniae* grown in LB were sensitive to dispersal by GH, whereas SVAM-grown *K. pneumoniae* biofilms were impervious to dispersal by GH. In the case of *C. albicans*, growth in YPD established a biofilm sensitive to DNase but insensitive to GH, whereas biofilms established in SVAM were glycoside hydrolase sensitive but recalcitrant to DNase treatment. The susceptibility of *K. pneumoniae* biofilms, *P. aeruginosa* biofilms and *C. albicans* to proteinase K dispersal was similar irrespective of the growth medium used to establish the biofilm. Thus, the macromolecular composition of the extracellular matrix differs in biofilms established in SVAM.

### Combining antimicrobials with matrix-degrading enzymes can increase the eradication of biofilms grown in the IVETT model

**Fig. 6.**
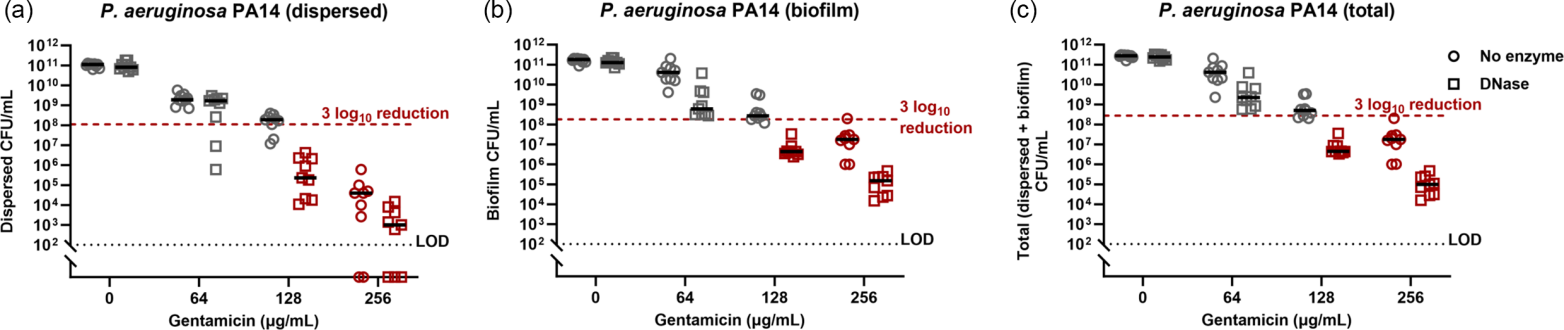
Treating *P. aeruginosa* grown in the IVETT biofilm model with a combination of DNase and gentamicin. Biofilms of *P. aeruginosa* were established on segments of a serum-coated endotracheal tube in SVAM for 48 h. Endotracheal tubes were then transferred to fresh medium and treated with either 100 µg ml^−1^ DNase or differing gentamicin concentrations for a further 24 h. Endpoint c.f.u.s of the dispersed bacteria in the surrounding medium (**a**), the endotracheal tube-associated biofilm (**b**) and the total population (dispersed + biofilm) (**c**) were acquired. The red dotted line denotes the 3 log_10_ reduction threshold. The black dotted line denotes the LOD and *n* = 3 biological repeats, with three technical repeats for each biological replicate.

We sought to evaluate combined treatments with matrix-degrading enzymes and antimicrobial drugs to overcome the antimicrobial tolerance in the endotracheal tube biofilms. There is clinical potential for such approaches, with recombinant human DNase (rhDNase) already in use as a mucolytic therapy in the airways of cystic fibrosis patients [63,64]. Thus, we investigated the efficacy of DNase in conjunction with gentamicin to treat *P. aeruginosa* biofilms. While DNase had little impact alone in enzymatic dispersal experiments (Fig. 5a), its presence alongside gentamicin had a synergistic effect on the eradication of *P. aeruginosa*: DNase plus 128 µg ml^−1^ gentamicin was better at reducing the total viable *P. aeruginosa* population than either 128 µg ml^−1^ or 256 µg ml^−1^ gentamicin alone (Fig. 6c). Importantly, this treatment killed cells in both the dispersed (Fig. 6a) and biofilm (Fig. 6b) populations of *P. aeruginosa*, showing that this combination did not simply disperse the biofilm; it also improved killing by gentamicin.

**Fig. 7.**
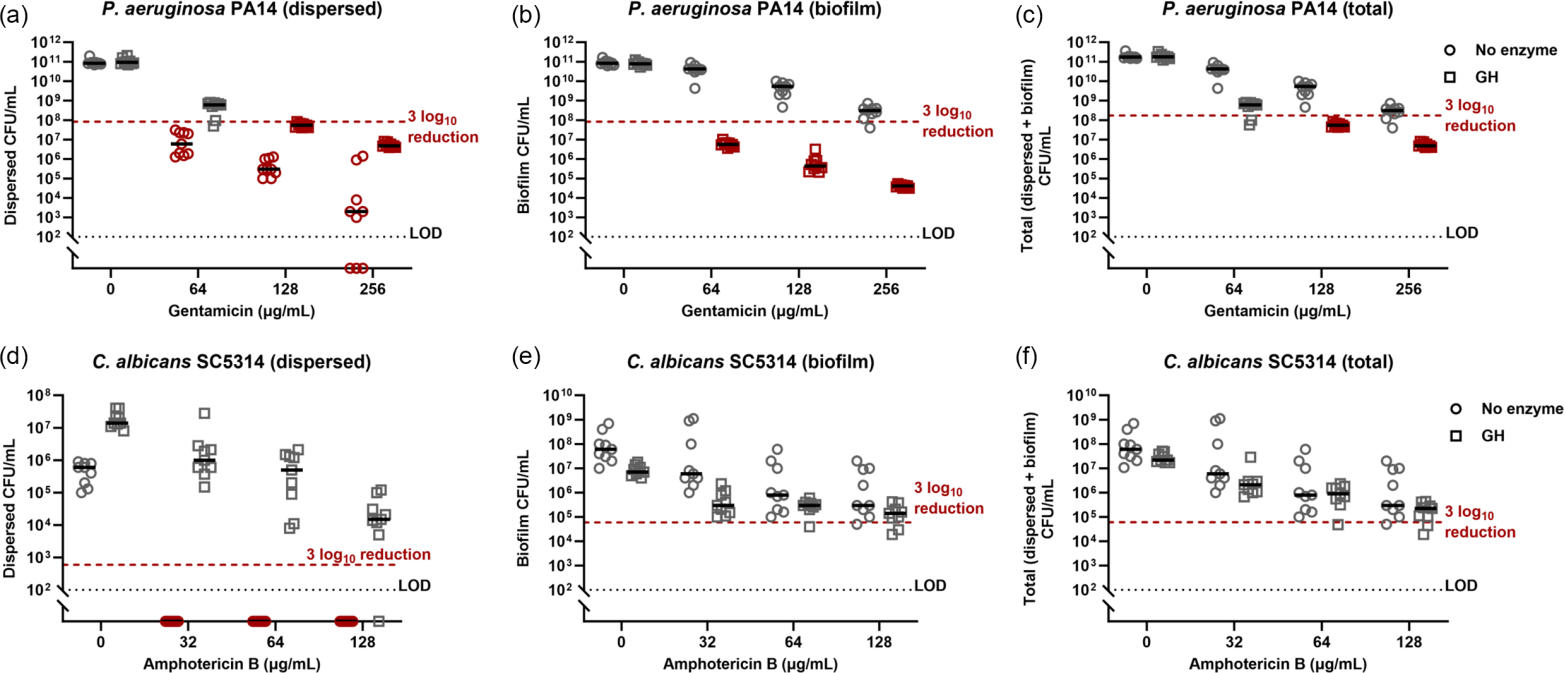
Treating *P. aeruginosa* and *C. albicans* with a combination of GH and antimicrobials. Biofilms of *P. aeruginosa* or *C. albicans* were grown on segments of serum-coated endotracheal tube in SVAM for 48 h. Endotracheal tubes were then transferred to fresh medium and treated with either 10% GH and differing concentrations of either gentamicin (*P. aeruginosa*) or amphotericin B (*C. albicans*) for a further 24 h. Viability of *P. aeruginosa* was determined by acquiring the endpoint c.f.u.s of the dispersed (**a**) and biofilm (**b**), which when added together provide the total viability of *P. aeruginosa* (**c**). The viability of *C. albicans* was determined by acquiring the endpoint c.f.u.s of the dispersed (**d**), biofilm (**e**), which added together provide the total viability of *C. albicans* (**f**). The red dotted line denotes the 3 log_10_ reduction threshold. The black dotted line denotes the LOD and *n* = 3 biological repeats, with three technical repeats for each biological replicate.

GH significantly dispersed both *P. aeruginosa* and *C. albicans* endotracheal tube biofilms grown in SVAM (Fig. 5a and c). Therefore, we evaluated the effect of combining GH with gentamicin (*P. aeruginosa*) or amphotericin B (*C. albicans*). In both species, glycoside hydrolase treatment resulted in a higher viable dispersed population relative to treatment with antimicrobials alone (Fig. 7a and d). This was particularly notable for *C. albicans* biofilms, in which no viable fungi were detected in the dispersed population when treated with amphotericin B alone. In the case of combination-treated *P. aeruginosa,* this increase in the planktonic population was countered by a decrease in the viable biofilm population (Fig. 7b). This led to a 3 log_10_ reduction in viability of the total population being reached at 128 µg ml^−1^ and 256 µg ml^−1^ gentamicin when combined with glycoside hydrolase treatment, which was not achieved with the same concentrations of gentamicin alone (Fig. 7c). Glycoside hydrolase and amphotericin B resulted in minor decreases in the viability of *C. albicans* biofilm populations (Fig. 7e). This, taken alongside the increase in planktonic *C. albicans* when exposed to glycoside hydrolase, meant that none of the tested treatments achieved biofilm eradication in the total population (Fig. 7f). Treatment of *K. pneumoniae* biofilms with colistin and GH was not explored because *K. pneumoniae* biofilms were not dispersed by GH (Fig. 5b).

**Fig. 8.**
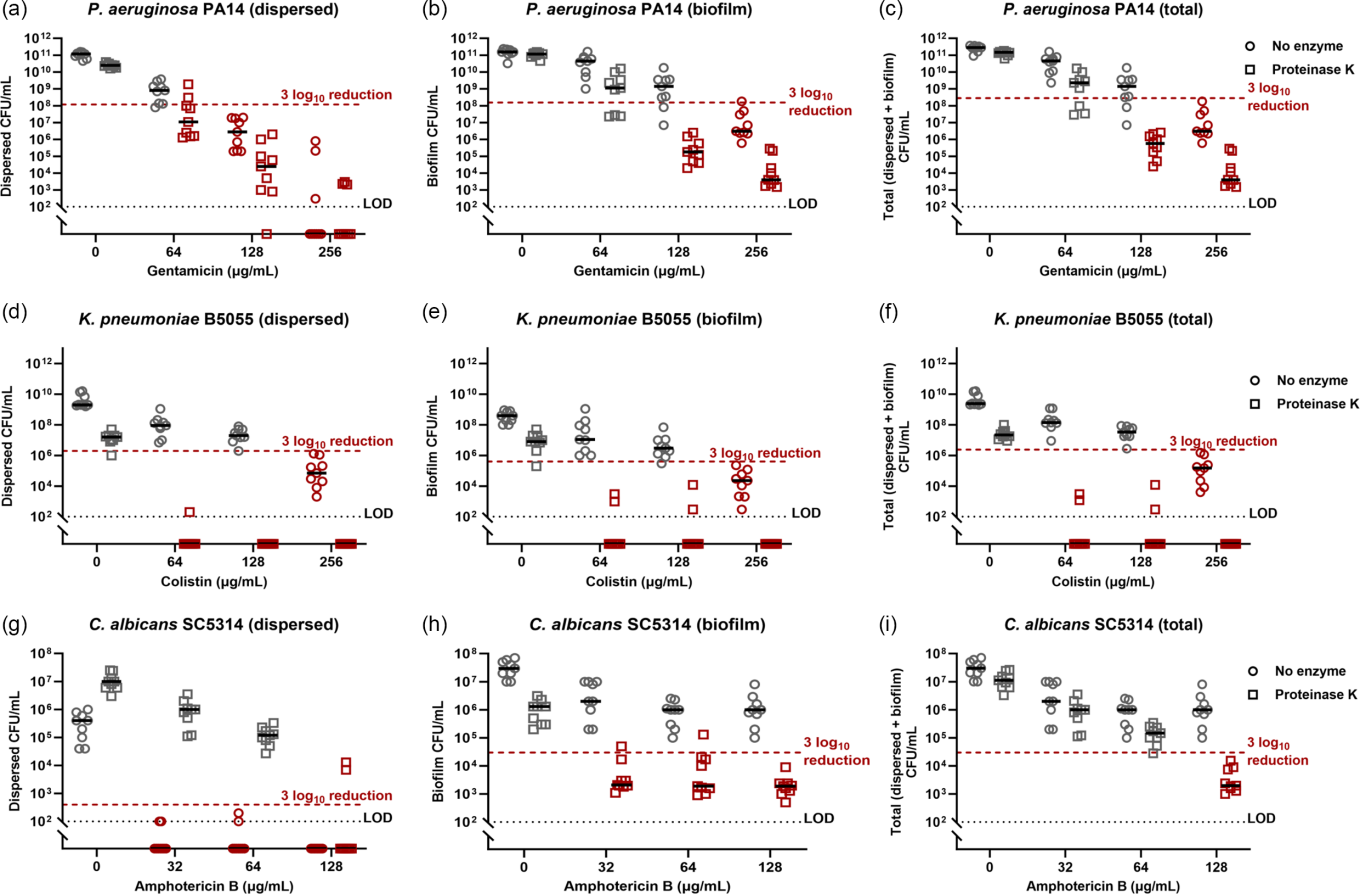
Treating biofilms grown in the IVETT biofilm model with a combination of proteinase K and clinically relevant antimicrobials. *P. aeruginosa*, *K. pneumoniae* or *C. albicans* biofilms were grown on segments of serum-coated endotracheal tube in SVAM for 48 h. Endotracheal tubes were then transferred to fresh medium and treated with either 1 mg ml^−1^ proteinase K and differing concentrations of either gentamicin (*P*. aeruginosa), colistin (*K. pneumoniae*) or amphotericin B (*C. albicans*) for a further 24 h. The viability of *P. aeruginosa* was determined by acquiring the endpoint c.f.u.s of the dispersed cells (**a**) and remaining biofilm (**b**), which when added together provide the total viability of *P. aeruginosa* (**c**). The viability of *K. pneumoniae* (**d–f**) and *C. albicans* (**g–i**) was similarly assessed. The red dotted line denotes the 3 log_10_ reduction threshold. The black dotted line denotes the LOD and *n* = 3 biological repeats, with three technical repeats for each biological replicate.

Proteinase K in combination with antimicrobials was the most effective treatment for both dispersing and eradicating biofilms of all species. The enzyme was most potent against *K. pneumoniae* when combined with colistin. This combination achieved almost complete eradication of dispersed (Fig. 8d), biofilm (Fig. 8e) and the total population (Fig. 8f) in the endotracheal tube model. Thus, the combination of 64 µg ml^−1^ colistin and proteinase K was far superior to even 256 µg ml^−1^ colistin alone. Combining proteinase K with gentamicin enhanced clearance of both dispersed (Fig. 8a) and biofilm-associated (Fig. 8b) *P. aeruginosa* compared to gentamicin alone. As with both DNase and glycoside hydrolase combination treatment, eradication of total * P. aeruginosa* was achieved by combining 128 µg ml^−1^–256 µg ml^−1^ gentamicin with proteinase K (Fig. 8c). As observed with glycoside hydrolase combination treatment, proteinase K treatment increased the recovery of dispersed *C. albicans* (Fig. 8g). However, unlike glycoside hydrolase combination treatment, proteinase K combined with amphotericin B led to a 3 log_10_ reduction in *C. albicans* biofilm viability relative to treating with amphotericin B alone (Fig. 8h). We observed that 128 µg ml^−1^ amphotericin B in conjunction with proteinase K eradicated both the dispersed and biofilm *C. albicans* populations (Fig. 8i).

## Discussion

VAP is broadly defined as pneumonia occurring in a patient who has been on mechanical ventilation for more than 48 h. VAP mortality rates can reach 45% [65,66]. However, widespread differences in VAP definition, diagnosis and sampling account for large discrepancies in VAP incidence and mortality rates globally [25,65,69]. The dangers of VAP have been highlighted over recent years due to the COVID-19 pandemic, in which many hospitalized COVID-19 patients required long-term mechanical ventilation [28]. Diverse microbial communities and complex biofilm structures have been found on the endotracheal tubes of mechanically ventilated COVID-19 patients [27,70]. The reported incidence of VAP in COVID-19 patients is much higher (50–80 %) [71] compared to non-COVID-19 patients (5–40 %) [25,72]. Despite the need to study VAP aetiology being higher than ever, the extensive development of host-mimicking media and *in vitro* and *ex vivo* infection models seen for conditions such as cystic fibrosis [40,47] has not transpired for VAP, restricting VAP microbiology research and ultimately limiting our ability to combat the endotracheal tube biofilms that promote this disease.

Here, we present a novel IVETT biofilm model, consisting of serum-coated endotracheal tube sections and a newly formulated growth medium to simulate the airway mucus of mechanically ventilated patients. We used this model to gain insights into the biofilm structure of three important VAP pathogens and demonstrated that the model can be used to screen candidate therapies to combat endotracheal tube biofilms.

Endotracheal tubes are readily available for laboratory use, and serum-coating them ensured that the substrate in our model simulated the conditioning layer that coats endotracheal tubes following intubation [73]. This layer is important for providing initial anchorage points to which microbes adhere. In medical device biofilms, this layer consists of proteins such as fibronectin and laminin and polysaccharides [74], all of which are present within FBS [75]. Our SVAM medium was designed following an extensive review of published literature [62]. It contains many components absent from standard laboratory growth medium such as mucin, DNA, metals, GlcNAc, albumin and lysozyme which provide nutrition or alter the gene expression of microbial pathogens in the pulmonary inflammatory disease environment [76,83] and induce antimicrobial tolerance [78,84,88]. While this model mimics the material and nutritional environment of ventilated airways, other aspects of the environment, such as the presence of flow and host cells are neglected. Adapting this model to include flow and intact endotracheal tubes would provide more realistic biofilm formation but may compromise the high-throughput nature of the model in its current form. Including *ex vivo* pig tissue alongside endotracheal tube segments may provide an ethical, easy-to-replicate method of including biological components present in ventilated airways. In the *ex vivo* pig lung cystic fibrosis model, this has been shown to provide biofilm structures and gene expression profiles that more closely replicate those seen in the host [46,49,89].

The biofilms established by ventilator-associated pathogens were more difficult to eradicate when grown in our model than in a Calgary device, even if SVAM was the growth medium in the Calgary device. This mirrors previous findings: biofilms grown in our *ex vivo* cystic fibrosis biofilm model were more tolerant to colistin treatment than biofilms grown in cystic fibrosis-mimicking medium in a Calgary device [3]. This increase in tolerance could be attributed to the serum-coated endotracheal tube upon which biofilms formed, as serum itself can induce antimicrobial tolerance [90].

From cryo-SEM of endotracheal tube biofilms, it was apparent that the model environment yielded very thick biofilm structures coated in a thick matrix. This is consistent with *in vivo* data in other studies that have found up to 84% of endotracheal tubes extubated from patients were completely covered in confluent biofilm [91], and that this high coverage of biofilm increased the likelihood of VAP [26]. The microcolony structures observed in our biofilms are similar to those formed in sputum due to the presence of mucin and DNA [92] and match the mushroom-like biofilms previously observed on the endotracheal tubes of mechanically ventilated COVID-19 patients [70]. Also, the overall structure of the *P. aeruginosa* biofilms in our model – punctuated with gaps, giving a lacy or spongy appearance – resembled that seen in a cystic fibrosis lung biofilm model using light microscopy [47,93, 94]. For LB-grown *K. pneumoniae*, only small aggregates of biofilm could be found on endotracheal tubes, while SVAM resulted in extensive, matrix-covered biofilms being observed. The rough cell surface of *K. pneumoniae* seen in SVAM is consistent with previous literature showing capsular polysaccharide on the cell surface of hypermucoviscous strains [95], such as *K. pneumoniae* B5055. The increased capsular polysaccharide in SVAM-grown biofilms might be ascribed to the presence of glucose and iron in the SVAM medium, both of which are known to impact capsular polysaccharide biosynthesis in *K. pneumoniae* [96,97].

The fungal pathogen *C. albicans* also displayed obvious morphological differences when grown in different endotracheal tube culture environments, favouring hyphal formation in YPD while remaining in yeast form or forming pseudohyphae in SVAM. GlcNAc, a component of SVAM, is known to induce filamentation in *C. albicans* [98]. Serum, which coats the endotracheal tubes for both the YPD and SVAM conditions, is also known to induce hyphal formation [99]. However, the reason this may not occur in SVAM could be due to the presence of high concentrations of mucin, which can suppress filamentation even in the presence of other hyphal-inducing conditions [100]. Hyphal formation is associated with increased virulence of *C. albicans*, while the yeast form is generally more associated with benign commensalism [101]. It would be interesting to understand if the distinct morphology and structure of endotracheal tube biofilms contribute to the rare association of * C. albicans* pneumonia in ventilated patients, despite its frequent isolation from endotracheal tube biofilms [20,23, 26, 102].

Since the biofilm matrix produced by VAP pathogens varied depending on the growth environment, matrix-degrading enzyme susceptibility was also seen to be altered. Matrix-degrading enzymes could be powerful adjuvants to increase the ability of antimicrobials to eradicate biofilms if the correct enzymes are chosen to ensure both dispersal and killing [103,104]. For example, DNase + gentamicin was tested for its ability to clear *P. aeruginosa* biofilms, as rhDNase is commonly prescribed for mucus clearance in the cystic fibrosis airway, an environment in which *P. aeruginosa* biofilms are a common affliction [63,64]. Alone, DNase did not significantly disperse *P. aeruginosa* biofilms as judged by crystal violet assays; however, DNase treatment did impact *P. aeruginosa* biofilms as it was seen to potentiate gentamicin killing of *P. aeruginosa* biofilms grown in the endotracheal tube model. Similar results of synergy have been reported for killing *P. aeruginosa* in cystic fibrosis sputum [105].

The glycoside hydrolase cocktail used for dispersal in this study consisted of α-amylase and cellulase, which hydrolyse α−1,4-glycosidic bonds and β−1,4-glycosidic bonds, respectively. These enzymes in conjunction with gentamicin have previously eradicated *P. aeruginosa* biofilms from murine chronic wounds [106], and this combination was also successful in our endotracheal tube model. While this glycoside hydrolase cocktail significantly dispersed *C. albicans* biofilms grown in the model, it did not increase biofilm eradication when used in combination with amphotericin B relative to amphotericin B alone. In the future, the use of β−1,3-glucanase to degrade β−1,3-glucan, one of the most important matrix polysaccharides in *C. albicans* biofilms, maybe a more appropriate candidate for testing [107,108]. Similarly, trials could be established with the use of bacteriophage-derived hydrolases that degrade the capsular polysaccharide of *K. pneumoniae* strains such as B5055 [109,110].

In our model, we assessed the viability of both the biofilm and dispersed populations after treatment with enzymes plus antimicrobials, as it is important to ensure that any biofilm dispersed from the endotracheal tube was sufficiently killed. Enzymatic degradation of biofilms without sufficient killing of dispersed cells could cause the dissemination of infection [111]. This is very important in the context of VAP, which results from the dispersal of biofilm deeper into the airways [19]. Tightly controlled dosage and administration of matrix-degrading enzymes and antimicrobial combinations, alongside the already routine regular suctioning of ventilated airways to clear mucus and dispersed pathogens [112], would be necessary if combination treatment were to be tried clinically.

In conclusion, through the development of SVAM growth medium combined with serum-coated endotracheal tube sections, we have produced an *in vitro* model that mimics the material, chemical and nutritional environment surrounding ventilator-associated pathogens. As seen *in vivo*, we observed elevated antimicrobial tolerance and dense biofilm formation. As proof of concept that the model can be used to screen novel therapies, we assessed the ability of selected matrix-degrading enzymes to enhance the antibiofilm activity of clinically relevant antimicrobials. Some combinations, particularly proteinase K and colistin, were effective at eradicating both dispersed and endotracheal tube biofilm populations from the ventilator-associated environment. In the future, this IVETT model can be used to conduct more detailed explorations of ventilator-associated pathogen biology, identify novel drug targets and screen candidate drugs or antimicrobial endotracheal tube coatings for likely efficacy before proceeding into *in vivo* trials.

## Abbreviations

CaMHB: cation-adjusted Mueller Hinton broth
COVID-19: coronavirus disease 2019
cryo-SEM: cryo-scanning electron microscopy
EUCAST: European Committee on Antimicrobial Susceptibility Testing
GH: glycoside hydrolases
IVETT: *in vitro* endotracheal tube
LB: lysogeny broth
LOD: limit of detection
MBEC: minimum biofilm eradication concentration
rhDNase: recombinant human DNase
SVAM: synthetic ventilated airway mucus
VAP: ventilator-associated pneumonia
YPD: yeast extract peptone dextrose
